# A Comparative Study on the Muscle and Gut Microbiota of *Opsariichthys bidens* from Rice Field and Pond Culture Breeding Modes

**DOI:** 10.3390/metabo14080443

**Published:** 2024-08-09

**Authors:** Fan Zhou, Weichao Bu, Hongjie Fan, Shuirong Guo, Ming Qi, Gaohua Yao, Yijiang Bei, Yuanfei Huang, Shicheng Zhu, Xueyan Ding, Xingwei Xiang

**Affiliations:** 1 Zhejiang Fisheries Technical Extension Center, Hangzhou 310012, China; zhoufan0302@126.com (F.Z.); qiming_1120@163.com (M.Q.); 15988855806@163.com (G.Y.); zsbij@163.com (Y.B.); 13567189845@163.com (Y.H.); 2 College of Food Science and Technology, Zhejiang University of Technology, Hangzhou 310014, China; 221122260061@zjut.edu.cn (W.B.); 19858106237@163.com (H.F.); zhusc@zjut.edu.cn (S.Z.); 3 Hangzhou Center for Agricultural Technology Extension, Hangzhou 310020, China; xsgsr@163.com

**Keywords:** meat quality, *Opsariichthys bidens*, pond culture, rice field culture, nutritional quality

## Abstract

To investigate difference in the quality of the different parts (back, tail muscles, and fish skin) of *Opsariichthys bidens* from pond and rice field cultures, a comparative study was conducted in terms of nutritional composition, volatile flavor profiles and gut microbiota. In detail, the texture, free amino acids, fatty acids were further assessed. The results suggested that the moisture content, crude protein and crude fat content in the skin of *O. bidens* are higher than those in the back and tail muscles, regardless of breeding modes. The fish cultured in the rice field had a higher protein content than those from the pond culture, while the fat content of the rice field-cultured fish was significantly low compared to the fish from the pond culture, especially in the back and tail parts. A total of 43 volatile components were detected by Gas Chromatography–Mass Spectrometry (GC-MS), with a maximum of 18 types of aldehydes and the highest concentration being nonanal. Compared to pond cultures, the fish from the rice field cultures showed more abundant flavor composition and odor-active compounds. The total content of DHA (Docosahexaenoic Acid) and EPA (Eicosapentaenoic Acid) in the rice field-cultured fish was higher than that of the pond group, while no significant disparity in amino acid composition was observed (*p* > 0.05). Comparative and clustering analyses of gut microbiota revealed notable discrepancies in the gut microbiota of *O. bidens* from two aquaculture systems. However, an inherent correlation between the gut microbiome and meat quality would be further emphasized in further studies. This study can offer a theoretical reference for the development of high-quality aquatic products by selecting the appropriate aquaculture models.

## 1. Introduction

Pond aquaculture is poised to emerge as the primary production method in Chinese aquaculture. Nevertheless, the fast development of pond culture in China has caused a deterioration in water quality and frequent diseases, and even reduced production quality. Hence, these serious challenges to the environment in pond aquaculture have forced us to explore sustainable aquaculture models [1]. The Rice–Fish System (RFS) is an integrated agricultural and aquaculture system (IAAS) combining rice cultivation and aquaculture, recognized as a “significant global agricultural heritage system” [2,3,4]. Compared to cultivating rice alone, RFS incorporates a diverse range of aquatic species [5,6]. RFS has the capability to collect durable aquatic products as well as rice, thereby accomplishing the objective of raising farmers’ income [7]. Hence, RFS aquaculture models have become popular in the regions of South China.

The interaction between rice and different animals co-cultivated within one system would maintain the equilibrium of the rice field ecosystem. The system can efficiently decrease the reliance on pesticides and fertilizers, as fish are able to consume pests and aquatic plants to safeguard rice, while their excretions can enhance soil fertility [8]. Several commercial fish species, such as *Cyprinus carpio* and *Carassius carassius*, are commonly utilized in RFS [9]. Paddy fields (pf) harbor a rich variety of aquatic animals and plants, offering a natural food source for both rice and fish including insects, weeds, large algae and phytoplankton. This natural feeding system significantly aids in pest and weed control [10,11]. Moreover, rice–fish cultivation allows for the conversion of food into biomass and waste, which is then recycled back into the environment, thereby enhancing rice production yields. Thereafter, the improved product quality, increased financial return, and reduced environment degradation of RFS contribute to providing high-quality aquatic products. Under the RFS mode, the comprehensive income per acre is 20–30% higher than the traditional single cultivation mode, and it can reduce nitrogen emissions by about 30% and phosphorus emissions by about 25%, effectively reducing the risk of eutrophication in water bodies.

*Opsariichthys bidens* is an exceptional omnivorous and partially carnivorous freshwater fish species that can be located in stream habitats in North Korea and Japan, with a predominant distribution in China [12,13]. At present, *O. bidens* is being farmed on a large scale in the Zhejiang Province. Due to its high nutritional value and unique flavor, *O. bidens* has transformed from a harmful local species to an emerging commercial fish species. Understanding the factors that affect the quality of *O. bidens*, such as muscle texture, taste, and nutritional composition, can help develop more precise breeding and fishing strategies, ensuring that the *O. bidens* supplied to the market have a superior quality. Exploring the effects of different growth environments, breeding methods and treatment methods on the mouthfeel of *O. bidens* can meet consumers’ pursuit of delicious aquatic products. Therefore, exploring the quality differences in *O. bidens* in pond and rice field cultures is necessary for the optimization of aquaculture models.

Microorganisms colonize the vertebrate gastrointestinal tract and play an important role in maintaining host growth and development [14,15], promoting intestinal cell proliferation [16] and nutrient absorption [17], enhancing immunity and health [18] and preventing diseases [19,20]. In addition, during host development, the gut microbiota can have a profound impact on the host by obtaining energy from the diet, conferring pathogen resistance and stimulating intestinal function and the development of the immune system [21,22]. Interestingly, recent studies have concluded that muscle quality is closely related with the composition of the gut microbiota. Therefore, maintaining the dynamic stability of microbial communities is essential to maintaining the intestinal health of fish, and even meat quality. Exploring the correlation of gut microbial with muscle quality may provide some suggestions for improving muscle quality by modulating functional gut microbial.

Fish skin can be processed into various delicious foods. For example, fried fish skin is a popular snack with a crispy texture; fish skin is also used in some dishes to enhance the richness and uniqueness of the taste. In this study, a comparative study was conducted in terms of nutritional composition and volatile flavor profiles to investigate the quality differences in the different parts (back, tail muscles and fish skin) between *O. bidens* in pond and rice field cultures. In particular, the proximate composition, texture, volatile compounds, free amino acids and fatty acids were assessed for the fish from two aquaculture models. Furthermore, the underlying mechanisms revealing the quality differences were explored by comparative and clustering analyses of gut microbiota. This study aimed to provide a theoretical reference for the development of high-quality aquatic products through the selection of appropriate aquaculture models.

## 2. Materials and Methods

### 2.1. Sample Collection

The *O. bidens*, with a body length of 14 ± 2 cm and a weight of 26 ± 2 g, were collected under different modes. The pond aquaculture fish were taken from the Zhejiang Provincial Aquatic Technology Promotion Station (Xiaoshan Base, Hangzhou, China), and the rice field aquaculture fish were taken from the Hangzhou Jiangdong Farm (Qiantang District, Hangzhou, China), with a cultivation period of six months. The two culture systems had the same stocking density of 3 fish/m^2^. Each culture system had three replicated ponds. The experimental fish from two different breeding methods were ice-transported to the College of Food Science and Technology at the Moganshan Campus of Zhejiang University of Technology (Huzhou, China) within 3 h. The slaughtered fish were washed with potable water to remove extraneous matter. Then, a mechanical deboner/mincer (Baader 601, Lubeck, Germany) was applied to separate the frames and muscle. The bone and skin from the frames were separated manually. This is the general method used for fish skin, which has been universally used in many previous reports [23,24]. This study was conducted according to the Guide for Laboratory Animals developed by the Ministry of Science and Technology (Beijing, China). The animal utilization protocol was approved by the Institutional Animal Care and Use Commitee of Zhejiang Fisheries Technical Extension Center, Hangzhou, China, on 26 May 2020 (approval number SYXK-ZHE-2020-0009).

### 2.2. Sample Preparation

The muscles and skin from various parts (back and tail) of the *O. bidens* were collected, cleaned and dried using absorbent paper to remove surface moisture. The samples were then packaged into sample bags and kept at −18 °C for future analysis of fundamental components (moisture, ash, crude protein and crude fat). The back muscles of the *O. bidens* from the different farming methods were cut into 1 cm × 1 cm × 1 cm pieces to be utilized for texture assessment.

### 2.3. Proximate Composition Analysis

The proximate chemical composition of moisture, total lipid, crude protein and total ash in all samples was analyzed following the guidelines of Chinese National Standards: GB5009.3-2016 [25], GB5009.4-2016 [26], GB5009.5-2016 [27], and GB5009.6-2016 [28], respectively.

### 2.4. Texture Properties Analysis

The texture instrument (TA. XT PlusC, Stable Micro Mystems, Godalming, UK) was used to measure the whole texture (hardness, elasticity, cohesion, adhesion, rupture, viscosity, chewiness and other parameters), according to the modified method of Kubraunal ASNNM, 2022 [29].

TPA (Texture Profile Analysis) test conditions: the P36R flat-bottomed cylindrical probe was used to conduct two compression TPA mode tests on the samples. The rate before the test was 3 mm/s, the test rate was 1 mm/s and the post-test rate was 5 mm/s. The compression degree was 50% and the interval retention time was 5 s. The data collection rate was 200 pps; each group of samples was measured at least 6 times and the average value was taken.

### 2.5. Volatile Compound Analysis

Accurately weighted minced fish meat samples (3.00 ± 0.01 g) were placed into a 15 mL headspace injections bottle. The activated 75 μm PDMS SPME (Polydimethylsiloxane solid phase microextraction) extraction head was positioned directly above the sample in the headspace bottle. The headspace bottle was submerged in a 60 °C water bath for extraction for 60 min. Once the extraction was finished, the extraction head was manually detached and inserted into the gas chromatography–mass spectrometry (Trace 1300-ISQ QD, Waltham, MA, USA) injection port for thermal analysis. Each group of samples was measured at least 3 times and the average value was taken.

Injection port settings: we opted for a non-split mode, set the temperature to 240 °C, established the non-split time at 1 min, set the cushion flow at 5 mL/min, utilized helium as carrier gas, and regulated flow rate to 1 mL/min.

GC conditions: We utilized a DB–5 MS elastic capillary column (60 m × 0.32 mm × 1 μm). The initial temperature was set at 40 °C and ramped up at a rate of 3 °C/min to reach 100 °C, further ramped up at 2 °C/min to 150 °C, then increased at 8 °C/min to 240 °C, followed by a 5 min hold period.

MS conditions: We used the Electrospray Ionization mode with the ion source temperature set to 250 °C, transmission line temperature at 250 °C, and a full mass scan range from 35 to 500 atomic mass units, with the interval set at 0.2 s.

Qualitative analysis: We automatically matched the spectra of volatile substances with those in the standard spectral library (NIST 2014 and Wiley9, NIST, Gaithersburg, MD, USA), and reported the identification results of the volatile substance only when the positive and negative matching degrees were both greater than 800.

Quantitative analysis: We added 100 μL of internal standard solution (2,4,6-trimethylpyridine, concentration 10^−5^ g/mL) to minced fish meat, and its concentration was calculated by calculating the ratio of peak areas of each volatile substance to the internal standard. This method belongs to the semi-quantitative method (the correction factor for each volatile substance to the internal standard is set to 1), and the specific calculation formula is as follows:

$$\mathrm{Volatile}\;\mathrm{concentration}\;(\mathrm{ng}/g)=\frac{A_{i}\times C_{std}\times V_{std}}{A_{std}\times M}\times 1000$$

where “A_i_” is peak area of volatile matter, “A_std_” is peak area of internal standard substance, “C_std_”is concentration of the internal standard added and “V_std_” is the volume of internal standard added. “M” is mass of fish sample added.

#### OAV (Odor Activity Value) Analysis

The OAV method was used to evaluate the contributions of volatile compounds, according to the following equation:

OAV_i_ = C_i_/OT_i_

where C_i_ is the compound concentration, and OT_i_ is the odor threshold of a compound in water. Volatile compounds with an OAV > 1 are considered significant contributors to the aroma characteristics.

### 2.6. Fatty Acids Analysis

Fat extraction: The fish flesh was blended in a chloroform–methanol mixture (Shanghai Titan Scientific Co., Ltd, Shanghai, China) with a 1:3 ratio and sealed in a glass container for 12 h. We then transferred the infused solution to a separating funnel, added a small portion of saturated sodium chloride solution (Shanghai Titan Scientific Co., Ltd, Shanghai, China) to the container above the separating funnel, and allowed it to settle for stratification before proceeding with filtration. The mixture was filtered under a separation funnel using a funnel and flask, and a small quantity of anhydrous sodium sulfate was introduced at the base of the filter paper before we initiated the filtration process. The filtered solution was condensed through rotary evaporation at 40 °C and 50 rpm/min using a rotary evaporator (RE2000A, Shanghai Yarong Biochemical Instrument Factory, Shanghai, China).

Methylation of fatty acids: We combined 0.1 g of fat extract with 1 mL of sodium hydroxide methanol solution (Sinopharm Chemical Reagent Co., Ltd, Shanghai, China) in a 15 mL centrifuge tube and placed it in a water bath at 80 °C for 5 min. Once cooled, 1 mL of boron trifluoride (Shanghai Titan Scientific Co., Ltd, Shanghai, China) and 0.1 mL of hydroquinone methanol solution were mixed and heated at 80 °C for 2 min. The 0.2 mL of saturated sodium chloride solution and 1 mL of N-hexane were mixed for 10 s. Approximately 0.5 g of anhydrous sodium sulfate (Hangzhou Jigong Biotechnology Co., Ltd, Hangzhou, China) was added into the supernatant. The supernatant was filtrated with a 0.22 μM organic microporous filter membrane for gas chromatography analysis. Each group of samples was measured at least 6 times and the average value was taken.

Injection port settings and MS conditions are the same as 2.5.GC conditions: A DB-5 MS elastic capillary column (60 m × 0.32 mm × 1 μm) was utilized. The initial temperature was set at 90 °C for 5 min, followed by an increase of 15 °C/min up to 200 °C, and was further increased at 1 °C/min up to 240 °C, and maintained for 10 min.

### 2.7. Free Amino Acid (FAA) Analysis

Amino acid hydrolysis: Take 2 g of fish meat and 15 mL of 15% TCA (trichloroacetic acid, Hangzhou Shuangmu Chemical Co., Ltd, Hangzhou, China) solution, and combine them in a 50 mL centrifuge tube. Homogenize for 10 s, then allow to settle for 2 h. Subsequently, centrifuge at 10,000 rotations per minute for 15 min in a centrifuge (ST16R, Thermo Fisher Scientific, Hangzhou, China). After centrifugation, take 10 mL of the supernatant and adjust the pH to 2.0 using a 1 mol/L NaOH (Sinopharm Chemical Reagent Co., Ltd, Shanghai, China) solution. Then, bring the volume up to 25 mL and filter through a 0.22 μM organic microporous filter membrane for analysis with an amino acid automatic analyzer (LA8080, Hitachi Ltd., Tokyo, Japan). Each group of samples was measured at least 6 times and the average value was taken.

### 2.8. Gut Microbe Analysis

Cut open the abdomen of the fish with a dissecting knife in a sterile environment, separate the intestinal contents, and place them in a 2 mL freezer tube. Store in a −80 °C refrigerator for later use. The DNA extraction and PCR amplification process was completed by Shanghai Major Bio-pharm Technology Co., Ltd. (Shanghai, China).

### 2.9. Statistical Analysis

**Figure 1:**
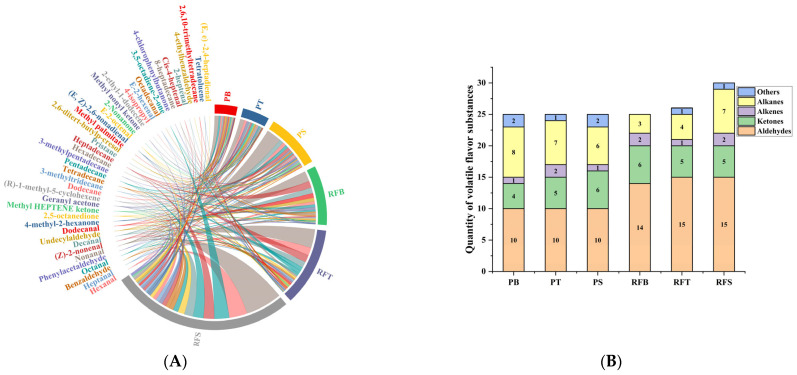
(**A**) Chord chart of the proportion of volatile components in *Opsariichthys bidens* under different cultivation modes; (**B**) quantity of volatile flavor compounds in *Opsariichthys bidens* under different cultivation modes. PB: back muscle of pond-cultured fish; PT: tail of pond-cultured fish; PS: skin of pond-cultured fish; RFB: back muscle of rice-bred fish; RFT: tail of rice-bred fish; RFS: skin of rice-bred fish.

**Figure 2:**
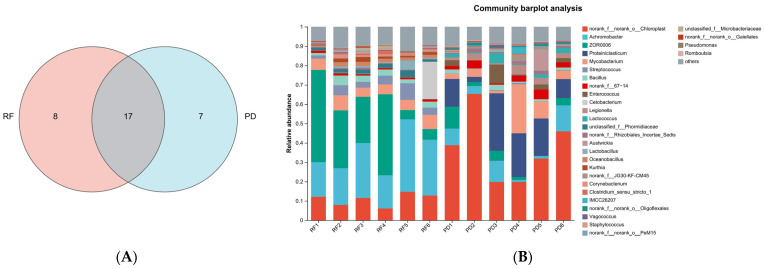
(**A**) Venn diagram of intestinal microflora of *Opsariichthys bidens* in two farming modes; (**B**) Community barbolt analysis of intestinal microflora in two farming modes of *Opsariichthys bidens.* RF: rice field aquaculture; PD: pond aquaculture.

**Figure 3:**
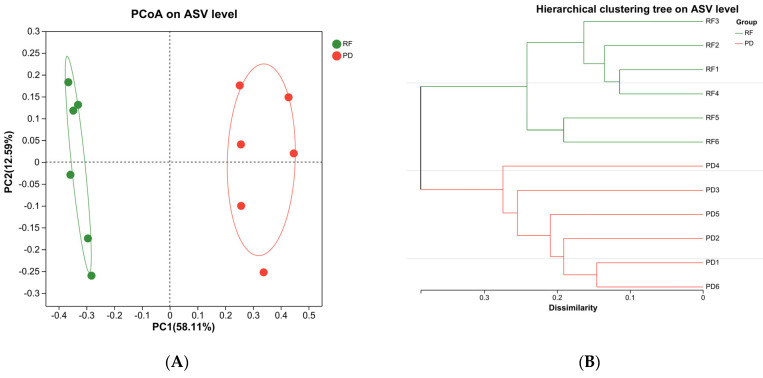
(**A**) Principal component analysis of intestinal microflora in two farming methods; PC1 (Principal Coordinate 1) and PC2 (Principal Coordinate 2) are used to describe the two main dimensions of differences and similarities between samples. (**B**) According to the hierarchical clustering tree of the two breeding modes, the samples are clustered according to the similarity, and the branch length between the samples is negatively correlated with the similarity. RF: rice field aquaculture; PD: pond aquaculture.

**Figure 4:**
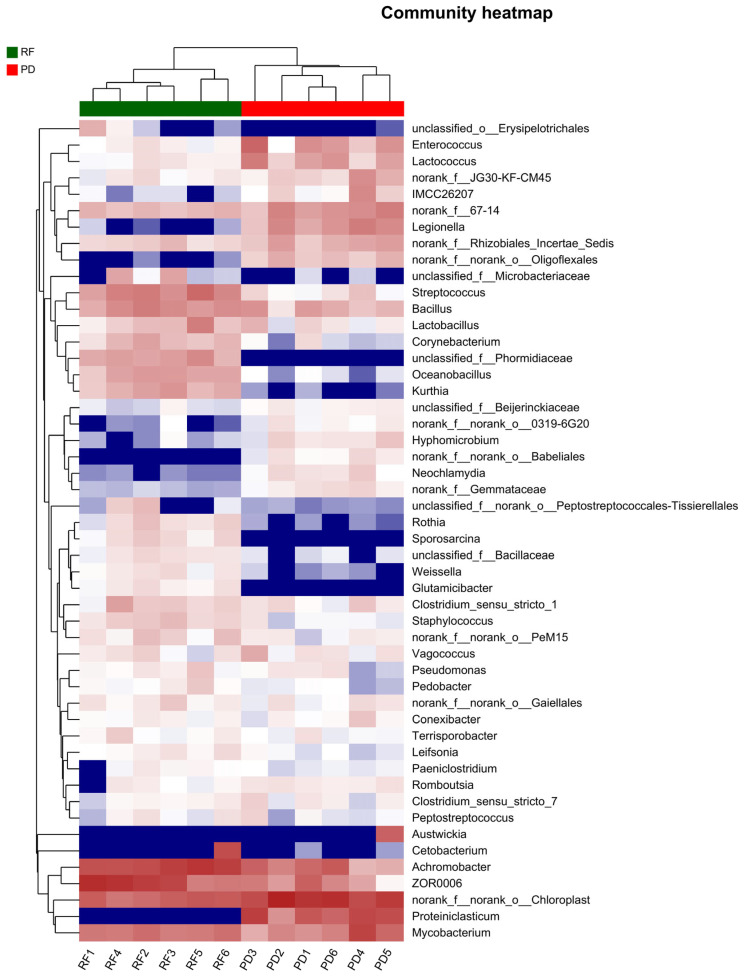
Two-level diagram of bacterial distribution in 12 samples. The heatmap represents the relative abundance of each bacterial genus (variables clustered on the vertical axis) in each sample (horizontal clustering). The values of bacterial genera are expressed as color intensity, as shown in the legend on the right side of the figure. RF: rice field aquaculture; PD: pond aquaculture.

Analysis was performed by one-way ANOVA using SPSS 23.0 (IBM Corporation, Armonk, NY, USA) with Duncan’s multiple range test, *p* < 0.05 indicates significant differences in the data. Figure 1 was plotted using Origin Pro 2021 (Origin Lab Corp., Northampton, MA, USA). Figure 2, Figure 3 and Figure 4 were created using the Majorbio website tool (QIIME2), URL (acceesed on 18 May 2024): https://cloud.majorbio.com/page/flow/index.html.

## 3. Results and Discussion

### 3.1. Proximate Compositions

From Table 1, it can be seen that there were no significant differences detected in the moisture content, crude protein content or crude fat content in the back and tail muscles of the *O. bidens* cultured under the two aquaculture modes (*p* > 0.05). However, the crude protein and crude fat content in the fish skin were significantly higher than those in the back and tail (*p* < 0.05). In general, the fish cultured in the rice field had a higher protein content than those cultured in the pond culture, while the fat content of rice field-cultured fish was significantly low compared to the fish from pond culture. The back muscles of the fish bred in the rice field almost decreased by 50% fat relative to the fish from pond culture, the differences in the skin of the fish from the two aquaculture modes are only reflected in water content, with no significant differences in crude protein content and crude fat content. The results suggested that altering culture modes can change proximate compositions of fish to some extent, which may lead to a distinctive quality performance.

### 3.2. Volatile Compound Composition

The HS-SPME-GC-MS (headspace solid-phase microextraction-gas chromatography–mass spectrometry) method was employed to analyze the volatile compounds in various parts of *O. bidens* under different farming conditions. A total of 18 aldehydes were detected in the volatile profile of *O. bidens*. Hexanal, Heptanal, Octanal, Nonanal, (Z)-2-nonenal, Decanal and Undecylaldehyde were identified in the back, tail and skin under both farming modes. Unique flavor compounds such as E-2-hexenal were found only in the rice field-cultured *O. bidens*, while Octadecanal was specific to the back muscles in rice cultivation. Cis-4-heptenal, 4-ethylbenzaldehyde, (E,E)-2,4-heptadienal and 2-heptenal were important flavor components in the tails and skin of the rice field-cultivated *O. bidens*. Additionally, eight ketones including Methyl heptanone and Geranyl acetone were detected in the back, tail and skin, while 2-nonone was a distinct flavor compound in the pond-cultivated *O. bidens*. Unique components like 3,5-octadiene-2-one and 4-chlorophenylbutanone were specific to paddy-cultivated *O. bidens*. Among the alkenes identified, 2-ethyl-1-dodecene was a characteristic volatile compound in the pond-cultivated *O. bidens*. Furthermore, the odor-active substance 8-heptadiene was detected in the back muscles, tail muscles and skin of the rice field-cultivated *O. bidens*. Unique alkanes such as 2,6,10-trimethyltetradecane were identified as active ingredients in the back and skin of *O. bidens* in rice fields. In the study, it was found that the concentration of fishy substances such as hexanal and (Z)-2-nonenal in the PD group was higher than that in the RF group. Previous studies have concluded that fishy substances are closely related to water environments [30]. It is speculated that the high content of nutrients and nitrogen and phosphorus elements in the water bodies used in the rice paddy farming mode is due to factors such as crop fertilization, which makes it more suitable for the large-scale reproduction of various planktonic algae. Therefore, the high content of fishy substances in the water environment used in rice paddy farming is convenient for the accumulation of more muscly fish substances. Most alcohols in this study were also observed in other aquatic animals [31,32,33]. The high thresholds of esters and alkanes indicated that these volatile compounds contributed slightly to the aroma [34]. In contrast, aldehydes are classified as the main flavor source because of their higher content and lower threshold than other volatile compounds [35]. The highest content of all substances, nonanal, has citrus, fatty and floral aromas, providing a refreshing sensory characteristic. The total concentration of volatile components in the RF group is significantly higher than that in the PD group, so the flavor of the RF group is richer than that of the PD group.

### 3.3. OAVs of Volatile Compounds

OAV can be used to assess the relative importance of individual volatile compounds to the odor profile of a food [36,37]. The volatile compounds with OAVs > 1 are considered relatively important compounds [30]. There are a total of seven types of odor-active compounds in the PD mode, while there are nine types in the RF mode (Table 2). The odor-active compounds in the MP sample provide a more rich flavor performance compared to PD, including E-2-hexenal, 4-ethylbenzaldehyde and 3,5-octadiene-2-one which are exclusively found in rice paddies. These compounds release pleasant odors such as fatty aromas, rich fresh fruit scents, green leaf fragrances and sweet tastes. Some microorganisms can sense the presence of 4-ethylbenzaldehyde and respond to it through specific signaling pathways. These odor-active compounds may affect the enzyme activity of microorganisms and alter the concentration of metabolites within microbial cells. For example, it may inhibit the activity of certain key metabolic enzymes, causing changes in the flux of related metabolic pathways, thereby affecting the growth rate and biomass accumulation of microorganisms. For microorganisms, 4-isopropyltoluene may interfere with their respiration or substance transport processes at certain concentrations. It may bind to receptors on the surface of microbial cells, affecting signal transduction and altering the composition of gut microorganisms [38,39]. On the contrary, the odor-active compounds of PD samples, like 2,6-di-tert-butyl-p-cresol and 4-isopropyltoluene, lack these aroma characteristics.

### 3.4. Texture Properties

As shown in Table 3, it can be seen that there are significant differences in the texture characteristics of *O. bidens* cultivated under different aquaculture modes. The hardness, adhesiveness, springiness and cohesiveness of the PB group are higher than those cultivated under RFB mode. From this, it can be seen that under the pond aquaculture mode, the *O. bidens* are chewier and have a tighter meat quality than those raised in rice paddies.

### 3.5. Fatty Acids Composition

As shown in Table 4, we found a total of twenty-nine fatty acids in *O. bidens* across the two farming methods. This includes ten saturated fatty acids (SFA), eight monounsaturated fatty acids (MUFA) and eleven polyunsaturated fatty acids (PUFA). The unsaturated fatty acid (UFA) content of 82.36% in the PD group was slightly higher compared to the RF sample. MUFA content was predominant in both farming methods at 46.67% and 51.84% respectively. The key unsaturated fatty acids in the two fish meat areC18:1n9c (about 20%) and C18:2n6c (about 26%), while the primary saturated fatty acid is C16:0 (about 11%).

According to nutritional classification, fatty acids that are crucial for development and health but cannot be synthesized by humans are considered essential fatty acids, while fatty acids that can be produced by humans are classified as non-essential fatty acids [40]. Recent studies have shown that omega-3 and omega-6 fatty acids can not only reduce the cardiovascular incidence rate, but also play an active role in the treatment of COVID-19 [41,42]. Studies have confirmed that DHA (Docosahexaenoic Acid) and EPA (Eicosapentaenoic Acid) have many functions, including benefiting cranial nerve development and reducing the risk of the risk of chronic illnesses such as cardiovascular diseases and diabetes, among others [43]. Sushchik et al. [44] have reported that the differences in living environment, food sources, seasons and activity spaces can affect the composition of fatty acids. The impact on PUFA is greater than SFA. The results from our study also show a similar performance. Although the total content of omega-3 and omega-6 fatty acids in PD is higher than that in RF, the fish from rice field aquaculture showed a higher content of EPA + DHA compared to the pond aquaculture.

### 3.6. Free Amino Acid (FAA) Composition

Table 5 displays the amino acid compositions of *O. bidens* farmed in ponds and rice fields. Aspartic acid (Asp) and glutamic acid (Glu) are essential FAAs that can synergize with nucleotides to enhance the freshness of fish [45]. ƩFAA is the basis for the taste presentation of aquatic animals, it is an important precursor for the formation of aromatic compounds [46]. A total of 17 different amino acids were identified, with histidine (His) being the most abundant (around 1.8 g/100 g) and valine (Val) the least (about 0.03 g/100 g). No significant differences in the types and quantities of amino acids between the two farming methods were found. The total amino acids (ƩAA), total free amino acids (ƩFAA), total essential amino acids (ƩEAA) and sweet amino acids were slightly high in the PB group, while minor variations were observed in fresh amino acids. The results suggested that altering aquaculture models may change amino acid composition, further affecting flavor characteristics, especially its freshness and sweetness.

### 3.7. Gut Microbial Community Analysis

#### 3.7.1. Analysis of Intestinal Flora Composition

It is evident from Figure 2A that the phylogenetic analysis of the sequences obtained in this research revealed 32 distinct phyla or groups, with 17 common species between the two farming practices. As shown in Figure 2B, we found that there are major phyla in the fish gut, such as Firmicutes, Proteobacteria, Actinobacteria, and Bacteroides. ZOR0006 and Acrobacter were predominant in the RF sample, while norank_f_ norank_o_ Chloroplast had a slightly lower abundance. In contrast, the PD sample showed a different trend, with norank_f_ norank_o_ Chloroplast being the most abundant, and ZOR0006 and Acrobacter decreasing significantly, replaced by an increase in Proteiniclastium. The combined abundance of these four microorganisms alone surpasses 80% of the total population, influencing the microbial variations between the two samples. This aligns with the disparities illustrated in the PCoA (Principal Coordinate Analysis) plot, indicating a notable distinction in the gut microbiota of *Opsariichthys bidens* under the two aquaculture modes.

#### 3.7.2. Sample Clustering Analysis

PCoA [47] analysis reveals the potential principal components influencing variations in sample community structure by reducing dimensions. The proximity and spread of the sample data on the two-dimensional coordinate map indicate the similarity level between samples. Figure 3A illustrates significant disparities in the composition of the two sample groups, with the two principal coordinates explaining 58.11% and 12.59% of the variance, respectively. Furthermore, hierarchical clustering analysis of microbial diversity at the genus level was conducted for various sample types [48]. The results showed that the aggregation distance of the two groups was relatively large, and the dominant microbial genera in each group were significantly different (Figure 3B).

#### 3.7.3. Core Gut Microbiota Analysis

Fish intestines harbor a diverse and rich microbiota, which establishes a complex and stable dynamic equilibrium with intestinal tissues and contents, contributing to intestinal development and health. The intestinal microbiota plays a crucial role in improving and maintaining the fish intestinal environment, promoting nutrient digestion, enhancing fish immunity and improving muscle quality. In the present study, the gut microbiota of *O. bidens* from two aquaculture systems almost aligned with earlier findings [49]. Firmicutes, Proteobacteria and Bacteroidetes always exist in the intestines of various fish, forming the core microbial community that effectively regulates the diversity and structure of fish gut microbiota. Certain microorganisms in the gut, such as *Bifidobacterium*, *Lactobacillus*, *Bac-teroidetes*, etc., can ferment dietary fiber to produce SCFAs (short chain fatty acids). The composition and diversity changes in microbial communities can affect the yield of SCFAs, and the level of SCFAs can in turn affect the growth and metabolism of microorganisms. SCFAs not only provide energy for intestinal epithelial cells, but also have functions such as regulating intestinal pH and inhibiting inflammatory reactions [50]. The abundance of bacteria in the phylum Proteobacteria affects the production of TMA (trimethylamine) and TMAO (trimethylamine oxide). TMAO levels are associated with cardiovascular disease risk, and changes in microbial communities may affect health by influencing TMAO production [51]. The analysis chart indicates a significant distribution discrepancy between the two sample groups, reflecting different culture environments and showing noticeable variations in gut microbiota composition. Hierarchical clustering analysis produced congruent outcomes. These variations might be attributed to the intricate and ever-evolving ecological surroundings in which the fish reside. Alterations in habitat, temperature, diet and gastrointestinal structure could impact the diversity of their gut microbiota [52]. Although this research examined the effects of different aquaculture methods on the intestinal microbiota of *O. bidens*, it is important to acknowledge that certain external factors, like water quality and diet composition, which were not thoroughly examined in our study, could also influence the outcomes. We remain dedicated to enhancing the experiment in future phases to account for the inherent correlation between the gut microbiome and meat quality.

## 4. Conclusions

The present study suggested that different aquaculture systems showed the distinct effects on meat quality of *O. bidens*. The fish cultured in rice field had a higher protein content than those from the pond culture, while the fat content of the rice field-cultured fish was significantly low compared to the fish from pond culture, especially in the back and tail parts. Compared to pond cultures, the fish from rice field cultures showed more abundant flavor compounds and higher content of EPA and DHA, while no significant disparity in amino acid composition was observed (*p* > 0.05). Comparative and clustering analyses of gut microbiota revealed notable discrepancies in the gut microbiota of *O. bidens* from the two aquaculture systems. However, the quantitative correlations between the gut microbiome and meat quality will be further emphasized in further studies.

## Figures and Tables

**Table 1:** Proximate composition of *Opsariichthys bidens* with the different parts between pond and rice field cultures.

Samples	Moisture (%)	Protein (%)	Fat (%)	Ash (%)
PB	77.65 ± 0.05 ^a^	15.99 ± 1.63 ^a^	2.81 ± 0.27 ^a^	1.05 ± 0.04 ^a^
PT	77.85 ± 0.72 ^a^	16.21 ± 1.16 ^a^	2.86 ± 0.01 ^a^	1.38 ± 0.02 ^a^
PS	61.85 ± 0.57 ^c^	18.50 ± 2.27 ^b^	3.34 ± 0.25 ^b^	1.06 ± 0.03 ^a^
RFB	78.16 ± 0.91 ^a^	17.60 ± 1.62 ^a^	1.57 ± 0.04 ^a^	1.13 ± 0.06 ^a^
RFT	78.11 ± 0.17 ^a^	17.84 ± 1.67 ^a^	1.67 ± 0.29 ^a^	1.20 ± 0.03 ^a^
RFS	64.50 ± 0.03 ^b^	19.70 ± 2.25 ^b^	3.50 ± 0.08 ^b^	1.12 ± 0.01 ^a^

Values are expressed as the means ± S.D. Different letters in same row indicate the significant different (*p* < 0.05). PB: back muscle of pond-cultured fish; PT: tail of pond-cultured fish; PS: skin of pond-cultured fish; RFB: back muscle of rice-bred fish; RFT: tail of rice-bred fish; RFS: skin of rice-bred fish.

**Table 2:** Odor activity values (OAVs) of volatile compounds of two kinds of culture modes of Opsariichthys bidens (muscles and fish skin).

NO.	Volatile Compound	CAS	Formula	Molecular Weight	Odor Threshold (ng/g)	OAV	
						PD	RF
	Aldehydes						
1	Benzaldehyde	100-52-7	C_7_H_6_O	106.120	41.7	>1	>1
2	Undecylaldehyde	112-44-7	C_11_H_22_O	170.300	140	>1	>1
3	Dodecanal	112-54-9	C_12_H_24_O	184.318	10	>1	>1
4	E-2-hexenal	6728-26-3	C_6_H_10_O	98.140	19.2	N.D.	>1
5	4-ethylbenzaldehyde	4748-78-1	C_9_H_10_O	134.180	123.23	N.D.	>1
6	(E,E)-2,4-heptane Olefinic aldehyde	4313-3-5	C_7_H_10_O	110.154	15.4	N.D.	>1
	Ketones						
7	2-Nonanone	821-55-6	C_9_H_18_O	142.239	38.9	>1	N.D.
8	3,5-octadiene-2-one	30086-02-3	C_8_H_12_O	124.180	150	N.D.	>1
9	4-chlorophenylbutanone	4559-96-0	C_10_H_10_BrClO	261.540	N.A.	N.D.	>1
	Others						
10	Tetradecane	629-59-4	C_14_H_30_	198.390	1000	>1	>1
11	2,6-ditert-butyl-p-cresol	821-55-6	C_9_H_18_O	142.239	1000	>1	N.D.
12	4-isopropyl toluene	99-87-6	C_10_H_14_	134.218	7.2	>1	N.D.

RF: Fish from rice field aquaculture; PD: Fish from pond aquaculture. “N.D.” and “N.A.” are abbreviations for “not detected” and “not acquired” respectively, representing data that is “not detected” or “cannot be obtained”.

**Table 3:** Texture properties of *Opsariichthys bidens* of different aquaculture modes.

	PB	RFB
Hardness (g)	1139.96 ± 232.77 ^a^	910.10 ± 236.58 ^b^
Adhesiveness	−13.73 ± 4.96 ^a^	−10.40 ± 1.12 ^b^
Springiness (mm)	0.53 ± 0.06 ^a^	0.49 ± 0.03 ^a^
Cohesiveness	0.51 ± 0.08 ^a^	0.34 ± 0.02 ^b^
Gumminess	578.54 ± 114.09 ^a^	309.20 ± 93.25 ^b^
Chewiness	305.94 ± 67.65 ^a^	148.98 ± 37.06 ^b^
Resilience	0.30 ± 0.03 ^a^	0.19 ± 0.01 ^b^

Values are expressed as the means ± S.D. Different letters in same row indicate the significant different (*p* < 0.05). PB: Back muscle of pond-cultured fish; RFB: back muscle of rice-bred fish.

**Table 4:** The fatty acid composition to total fatty acids in *Opsariichthys bidens* of different aquaculture modes (g/100 g).

Fatty Acids	Groups	
	PB	RFB
C12:0	0.02 ± 0.00	0.02 ± 0.00
C13:0	0.02 ± 0.01	0.02 ± 0.00
C14:0	1.25 ± 0.04	1.25 ± 0.12
C14:1n5	0.09 ± 0.00^a^	0.11 ± 0.01 ^b^
C15:0	0.34 ± 0.03	0.35 ± 0.01
C16:0	11.13 ± 0.09	10.64 ± 0.46
C16:1n7	7.58 ± 0.35 ^a^	9.23 ± 0.76 ^b^
C17:0	0.34 ± 0.03	0.36 ± 0.03
C17:1n7	0.53 ± 0.01 ^a^	0.64 ± 0.00 ^b^
C18:1n9t	6.06 ± 0.23	5.49 ± 1.99
C18:1n9c	20.74 ± 0.32 ^a^	24.75 ± 0.52 ^b^
C18:0	1.34 ± 0.28	2.41 ± 1.33
C18:2n6t	0.11 ± 0.02	0.11 ± 0.01
C18:2n6c	26.79 ± 0.27 ^a^	21.46 ± 0.20 ^b^
C18:3n3	0.68 ± 0.03	0.66 ± 0.03
C18:3n6	2.73 ± 0.01 ^a^	2.30 ± 0.05 ^b^
C20:0	0.17 ± 0.01	0.18 ± 0.01
C20:1	1.14 ± 0.03 ^a^	1.44 ± 0.06 ^b^
C20:2	0.88 ± 0.02 ^a^	0.79 ± 0.04 ^b^
C20:4n6	0.87 ± 0.01 ^a^	0.76 ± 0.05 ^b^
C20:3n6	1.70 ± 0.03 ^a^	1.60 ± 0.04 ^b^
C20:3n3	0.22 ± 0.02 ^a^	0.16 ± 0.02 ^b^
C22:0	2.99 ± 0.03	3.10 ± 0.14
C20:5n3	0.06 ± 0.01	0.06 ± 0.01
C22:1n9	0.03 ± 0.01	0.04 ± 0.02
C22:2n6	0.19 ± 0.02	0.20 ± 0.04
C22:6n3	1.45 ± 0.07 ^a^	1.70 ± 0.08 ^b^
C24:1n9	10.50 ± 0.35	10.13 ± 0.64
C24:0	0.04 ± 0.02	0.06 ± 0.01
EPA + DHA	1.51 ± 0.07 ^a^	1.76 ± 0.09 ^b^
SFA	17.64 ± 0.43	18.37 ± 1.22
MUFA	46.67 ± 0.29 ^a^	51.84 ± 1.60 ^b^
PUFA	35.69 ± 0.20 ^a^	29.79 ± 0.46 ^b^
UFA	82.36 ± 0.43	81.63 ± 1.22
omega-3	2.20 ± 0.09 ^a^	2.42 ± 0.12 ^b^
omega-6	27.67 ± 0.28 ^a^	22.22 ± 0.25 ^b^

Values are expressed as the means ± S.D. the different letters in same row indicate the significant different (*p* < 0.05). PB: back muscle of pond-cultured fish; RFB: back muscle of rice-bred fish.

**Table 5:** Free amino acid (FAA) composition of *Opsariichthys bidens* of different aquaculture modes (g/100 g).

Amino Acids	Groups	
	PB	RFB
Aspartic acid (Asp)	1.15 ± 0.33	1.20 ± 0.23
Threonine (Thr)	0.42 ± 0.11	0.43 ± 0.06
Serine (Ser)	0.34 ± 0.08	0.36 ± 0.05
Glutamic acid (Glu)	1.37 ± 0.23	1.32 ± 0.14
Alanine (Ala)	0.69 ± 0.20	0.81 ± 0.19
Glycine (Gly)	0.58 ± 0.16	0.62 ± 0.13
Valine (Val)	0.03 ± 0.00	0.04 ± 0.01
Cysteine (Cys)	1.27 ± 0.35	1.28 ± 0.25
Methionine (Met)	0.34 ± 0.09	0.34 ± 0.06
Isoleucine (Ile)	0.54 ± 0.16	0.55 ± 0.11
Leucine (Leu)	0.95 ± 0.28	0.97 ± 0.19
Phenylalanine (Phe)	0.37 ± 0.11	0.37 ± 0.07
Tyrosine (Tyr)	0.56 ± 0.16	0.57 ± 0.12
Lysine (Lys)	1.13 ± 0.34	1.16 ± 0.23
Histidine (His)	1.79 ± 0.54	1.85 ± 0.42
Arginine (Arg)	0.72 ± 0.21	0.76 ± 0.15
Proline (Pro)	0.45 ± 0.12	0.48 ± 0.11
Fresh Amino Acids	2.53 ± 0.55	2.51 ± 0.23
Sweet Amino Acids	1.61 ± 0.44	1.78 ± 0.36
ƩFAA	4.14 ± 0.99	4.29 ± 0.54
ƩEAA	6.29 ± 1.84	6.46 ± 1.31
ƩAA	12.70 ± 3.44	13.09 ± 2.30

PB: back muscle of pond-cultured fish; RFB: back muscle of rice-bred fish. Fresh amino acids: ASP + Glu; sweet amino acids: Ser + Gly + Ala; ΣFAA, flavor amino acids: fresh amino acids + sweet amino acids; ΣEAA, essential amino acid; ΣAA, total amino acids.

## Data Availability

The original contributions presented in the study are included in the article, further inquiries can be directed to the corresponding authors.
